# Cavity-Enhanced Raman Spectroscopy for Food Chain Management

**DOI:** 10.3390/s18030709

**Published:** 2018-02-27

**Authors:** Vincenz Sandfort, Jens Goldschmidt, Jürgen Wöllenstein, Stefan Palzer

**Affiliations:** 1 Laboratory for Gas Sensors, Department of Microsystems Engineering–IMTEK, University of Freiburg, Georges-Köhler-Allee 102, 79110 Freiburg, Germany; vincenz.sandfort@imtek.uni-freiburg.de (V.S.); jens.goldschmidt@imtek.uni-freiburg.de (J.G.); Juergen.Woellenstein@ipm.fraunhofer.de (J.W.); 2 Fraunhofer Institute for Physical Measurement Techniques IPM, Heidenhofstraße 8, 79110 Freiburg, Germany; 3 Department of Computer Science, Universidad Autónoma de Madrid, Francisco Tomás y Valiente 11, 28049 Madrid, Spain

**Keywords:** Raman spectroscopy, cavity, optical resonator, food chain management, ethene

## Abstract

Comprehensive food chain management requires the monitoring of many parameters including temperature, humidity, and multiple gases. The latter is highly challenging because no low-cost technology for the simultaneous chemical analysis of multiple gaseous components currently exists. This contribution proposes the use of cavity enhanced Raman spectroscopy to enable online monitoring of all relevant components using a single laser source. A laboratory scale setup is presented and characterized in detail. Power enhancement of the pump light is achieved in an optical resonator with a Finesse exceeding 2500. A simulation for the light scattering behavior shows the influence of polarization on the spatial distribution of the Raman scattered light. The setup is also used to measure three relevant showcase gases to demonstrate the feasibility of the approach, including carbon dioxide, oxygen and ethene.

## 1. Introduction

Even though the food industry is highly competitive and consequently cost-aware, the loss of fresh produce (vegetables and fruits) amounts to about 30% [1]. In order to improve this situation, monitoring technologies need to go beyond the currently employed temperature and humidity control [2,3,4]. In particular, the control of the gaseous atmosphere surrounding fresh food allows for inhibiting bacterial growth and tailoring the ripening process. To this end, nitrogen (N_2_), oxygen (O_2_), carbon dioxide (CO_2_), and ethene (C_2_H_4_) play a central role, since they may be used to regulate the aging process, prolong storage times, and deliver high quality products on-demand. So-called climacteric fruits, such as tomatoes and bananas [5], can be regulated well using environmental parameters [1,5,6]. In this case ethene may be used to control cellular respiration and consequently the oxygen usage and the associated release of carbon dioxide. Additionally, these fruits release ethene when ripening, which makes a control of the process paramount for long-term storage [7,8]. Consequently, improved food-chain management requires a miniature, in-situ sensorial system capable of quantitative and specific analysis of the complete chemical composition of surrounding air. Moreover, any suitable technology will need to be able to monitor the parameters across different chains, including production, packaging, and transportation. 

The analysis of a matrix of gaseous components along these chains is a complex task and ranges from the container gases used in transportation to prevent bacterial or vermin infestation [9] to simple molecules such as N_2_ or CO_2_ used in packaging. One standard method for monitoring gaseous concentrations in a cost-effective way are metal-oxide-(MOX)based gas sensors [10,11] or standalone flame ionization detectors [12]. Both show high sensitivity towards many gases, but have a poor performance in terms of selectivity. Even though it is possible to enhance the selectivity of MOX sensors using properties of the surface reactions, for example [13,14,15], or pattern recognition techniques [16,17,18], the technology is unsuitable for the selective detection of a large number of molecules. Another widely used technique is probing the molecular rovibrational absorption bands in the infrared regime. Techniques include nondispersive infrared spectroscopy (NDIR [19,20,21,22]) and tunable diode laser absorption spectroscopy (TDLAS [23,24,25,26]). Both are highly selective and may be tuned towards high sensitivities by enhancing the optical path length. Still they have the drawback of being cost intensive when measuring all components in a complex gas matrix. For example, to identify different gases with TDLAS, many wavelengths and therefore laser sources are needed. On the other hand, NDIR is limited by the need for many spectral filters. Fourier transform infrared spectroscopy (FTIR [27,28,29,30]) may be used to identify numerous different, infrared active components in a single measurement using only one spectrally broad light source. However, FTIR systems are expensive and bulky. The same is the case for the most commonly used methods for unknown gas matrices, i.e., gas chromatography (GC [31,32]) and mass spectrometry (MS [33,34,35]). 

On the other hand, spontaneous Raman spectroscopy offers the possibility to miniaturize multigas analysis using a single laser source. It is a well-known approach and widespread for liquids and crystals [36]. Due to the low-scattering cross section, it has been employed for gases less often [37]. Typical Raman scattering cross sections are many orders of magnitude below the absorption cross section in the mid-infrared region. For example, the Raman scattering cross section of carbon dioxide at 488 nm is around $\sigma_{Raman,CO2}=10.3\cdot 10^{-31}$ cm²/(mol·sr) ($1.9\times \sigma_{Raman,N2}$ [38]), compared to an absorption cross section on the order of 10^−19^ cm²/mol in the mid IR range at 4.2 µm [39]. In order to increase the Raman intensity, several levers may be used [40,41,42]:

(1) $$I_{Raman}=\;\varepsilon \cdot I_{Laser}\cdot \frac{N}{V}\cdot l_{eff}\cdot \sigma_{Raman\;},$$

where ε is the total detection efficiency, $I_{Laser}$ the excitation laser intensity, *N* the number of molecules in the illuminated volume *V*, $l_{eff}$ the effective interaction length and $\sigma_{Raman}$ the Raman scattering cross section. Obvious possibilities include an increase in laser intensity used to generate Raman scattered photons or decreasing the excitation wavelength $\lambda_{0}$, since the Raman scattering cross section behaves according to $\;\sigma_{Raman}\left(\lambda_{0}\right)\;\sim \;{\left(\frac{1}{\lambda_{0}}\right)}^{4}$, which in turn may be used to enhance the Raman scattering cross section. 

Lately a growing number of techniques to enhance Raman scattering have been employed. One way is surface-enhanced Raman spectroscopy (SERS [43,44,45,46]), which uses the electric field interaction between the probe material and metallic surfaces to enhance the effective Raman scattering cross section. In fact, various enhancement mechanisms may be deployed at once to boost Raman photon generation by as much as 10^10^ [46]. An increased $\sigma_{Raman}$ may also be achieved by using nonlinear techniques such as stimulated Raman spectroscopy (SRS [47,48,49]) and coherent anti-Stokes Raman spectroscopy (CARS [47,50,51]). Both yield an improvement of several orders of magnitude, yet they need a second, tunable laser source resulting in higher costs and more delicate setups with a limited frequency range. A third option is to enhance the effective interaction length $l_{eff}$ to improve the Raman scattered intensity with a single laser source to enhance the application range. One option to achieve this are capillary fibers [52,53], that keep an otherwise divergent beam inside a fiber and collect the scattered light. Fiber-enhanced Raman spectroscopy (FERS [54,55,56,57,58]) is working on the same basis: A hollow-core photonic crystal allows for low-loss guidance by creating a photonic band gap guidance.

Then there are techniques to enhance the laser light intensity, using a double reflection, multiple reflection cells [59,60,61], as well as cavity-enhanced Raman spectroscopy (CERS [62,63,64]), which multiplies the laser intensity inside an optical resonator build by two or more highly reflective mirrors. A possible setup was described by J. J. Barrett et al. in 1968, who moved a mirror of an ion laser and put the Raman sample into the laser cavity [65]. This was soon followed by G. O. Neely et al. in 1972 and R. S. Hickman and L. Liang in 1973 [66,67], who named the technology Intracavity Raman spectroscopy. In 2003 Ohara et al. introduced a similar mechanism with a laser diode [68], coupling an antireflection coated diode laser to a Fabry–Pérot cavity. The external cavity was used as a frequency standard and for intensity enhancement at the same time. Salter et al. then presented in 2012 a CERS technique [63], where the laser wavelength is modulated periodically with a saw-tooth waveform. The duty cycle of resonance is given as approximately 50%. Using an active stabilization to keep the optical cavity on resonance other groups have demonstrated continuous operation of CERS setups [69,70] in the past.

In this contribution a setup utilizing an actively stabilized optical cavity using the Pound–Drever–Hall locking scheme to enhance the laser power and enable long-term stable operation is described and characterized. The optical setup is considerably less complex and more compact than previously presented approaches [69,70]. The Fabry–Pérot cavity has been designed such that its linewidth is slightly larger than the linewidth of the extended cavity diode laser used as pump source. The laser power is coupled into the resonator and intensity enhancement is achieved via a prolonged photon lifetime and multiple reflections [71]. Both the spatial intensity distribution of the pump light and the Raman scattered light are simulated. The setup is used to demonstrate background free, simultaneous measurement of all relevant gas components in the food supply chain of climacteric fruits and can even be easily widened to more gases of interest. As opposed to previous work employing optical feedback from a cavity [68], an active stabilization is used to enhance long-term stability and robustness. 

## 2. Methods 

### 2.1. Experimental Setup

A home-built external cavity diode laser (ECDL) based on the design by Ricci et al. [72] is used as light source. It employs a grating stabilized, single mode laser diode (RLT785-150MGS, Roithner Lasertechnik GmbH, Vienna, Austria) with an elliptical beam shape at an aspect ratio of 1:1.9 and linear polarization with the electric field vector ${\tilde{E}}_{0}=\;A\left(\begin{array}{c} 0\\ 1 \end{array}\right)$ oscillating along the *y*-axis at a central wavelength of λ=780.2 nm. After passing a 60 dB optical Faraday isolator (I-80-U-4-L, Isowave Manufacturing, Dover, NJ, USA) the laser beam is separated with a 90:10 beam splitter into two parts. 10% of the laser power is used to perform Doppler-free absorption spectroscopy on a Rubidium vapor cell. Two cylindrical lenses with 20 mm and 38.1 mm focal lengths (LJ1328L2-B and LJ1765L1-B, Thorlabs, Newton, NJ, USA) change the diameter of the smaller axis of the laser beam to obtain a circular laser beam shape with a beam radius of $W_{l}=742$ µm. 

The optical cavity itself is formed by two identical plano-concave mirrors with 12.7 mm diameter, a concave curvature of $r_{C}=-500$ mm with a specified reflectivity $R_{HR}>99.85\%$, and a planar surface with antireflection coating (AR coating) with $R_{AR}<0.1\%$ (Laseroptik, Garbsen, Germany) at 780 nm, respectively. The mirrors are mounted on a custom made aluminium structure at a distance of ~38 mm, where one mirror is glued onto a hollow ring stack piezo allowing for fast adjustment of the distance between both mirrors. This forms a stable cavity with $g_{1}g_{2}=\left(1+\frac{L}{r_{c1}}\right)\left(1+\frac{L}{r_{c2}}\right)=0.8566$, i.e., well within the stability region stretching from 0 < $g_{1}g_{2}$ < 1. The theoretical frequency spacing $\nu_{FSR}$ (free spectral range) of the cavity’s ${\mathrm{TEM}}_{00}$-modes depends on the speed of light in the medium with refractive index *n* filling the cavity, i.e., $c=\;c_{0}/n$, and the distance L between the surfaces of the cavity mirrors [73]:

(2) $$\nu_{FSR}=\frac{c}{2\cdot L}\;.$$

As such, the free spectral range is expected to be about 3.945 GHz. If neglecting absorption and scattering losses in the gas filling, which is a reasonable assumption given that air has an attenuation factor of α=0.41 dB/km at 850 nm [74], the Finesse is governed by the mirror’s reflectivity R [73]:

(3) $$F=\frac{\pi \cdot \sqrt{\left|R\right|}}{1-\left|R\right|}\;.$$

**Figure 1:**
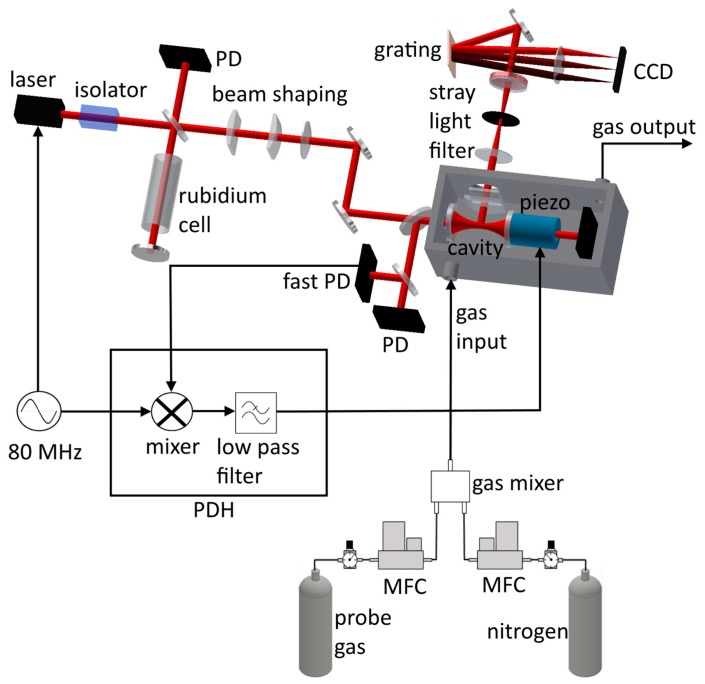
Schematic drawing of the cavity-enhanced Raman spectroscopy setup. An external cavity diode laser is guided through an optical isolator to the enhancement cavity, which in turn is stabilized with a Pound–Drever–Hall lock in back reflection. Raman scattered light is then collected with a detection optic, coupled through a stray light filter into a spectrometer and separated into the different wavelengths with a grating. Probe gas and nitrogen are controlled in their concentration with mass flow controllers (MFC). The two gases are then mixed in a gas mixer and guided to the flow box, containing the cavity.

Applying the manufacturer stated reflectivity of R≥0.9985, the Finesse is at least F≥2093. For F≫1 the linewidth Δν can be estimated by [73]:

(4) $$\Delta \upsilon \approx \;\frac{\nu_{FSR}}{F}\;,$$

resulting in a cavity linewidth of Δν=1.88 MHz. The estimated linewidth of the ECDL is well below 1 MHz [72], which should allow for coupling all available light into the Fabry–Pérot cavity. To check the absolute values of the free spectral range and the linewidth of the cavity, Doppler-free absorption spectroscopy of Rubidium is performed. For efficient optical coupling into a cavity a high overlap between the Gaussian eigenmode of the cavity (*res*) as well as the Gaussian beam of the laser is also necessary [71]. The beam parameters of the cavity eigenmodes are determined by the distance between the mirrors L and their respective radii of curvature R. For the symmetric spherical resonator used here the beam radius in the cavity’s center $W_{0,\mathrm{res}}$ and the Rayleigh range of this beam $z_{0,\mathrm{res}}$ read [73]:

(5) $$W_{0,\mathrm{res}}{}^{2}=\frac{\lambda \cdot L}{2\pi }\sqrt{2\frac{\left|R\right|}{L}-1}\;,$$

(6) $$z_{0,\mathrm{res}}=\frac{L}{2}\sqrt{2\frac{\left|R\right|}{L}-1}\;,$$

where λ is the exciting wavelength. For the employed cavity this results in $W_{0,\mathrm{res}}=154$ µm and $z_{0,\mathrm{res}}=95.6$ mm. Mode matching of the ECDL’s beam is achieved using a plano-convex lens with f = 500 mm which results in $W_{0,fl}=\;\frac{\lambda \cdot f}{\pi \cdot W_{l}}=166$ µm and $z_{0,fl}=\frac{W_{0,fl}^{2}\cdot \pi }{\lambda }\;=111$ mm for Gaussian beams which is in good agreement to the values inside the cavity. The complete optical setup for the cavity enhanced Raman spectroscopy setup is depicted in Figure 1.

The detection optics are placed in a 90°-geometry. Scattered light is collected with two cylindrical lenses featuring focal lengths of $f_{CL,1}=10$ mm and $f_{CL,2}=19$ mm, (LJ1878L2-B and LJ1095L1-B, Thorlabs) respectively. A stray light filter consisting of two lenses with 50 mm focal length (LA1131-B, Thorlabs) and a 300 µm wide pinhole is employed at a distance of 250 mm from the second cylindrical lens and directs the scattered light into a home-built spectrometer. It consists of a Raman edge filter (BLP01-785R-25, Semrock, Rochester, NY, USA), a grating with 1800 lines/mm, a focus lens with 80 mm focal length and a CCD line array (iDUS 401-BR-DD, Andor, Belfast, UK). The grating spectrally separates the incoming Raman scattered photons according to their wavelengths, which are then focused onto the CCD camera, having an image area of 26.6×3.3 mm² with 1024×127 pixels. The spectral range imaged on the camera ranges from ${\tilde{\nu }}_{low}=\tilde{\nu_{0}}-100$ cm^–1^ to ${\tilde{\nu }}_{high}={\tilde{\nu }}_{0}+3500$ cm^–1^ around the pump frequency at $\tilde{\nu_{0}}=12,816$ cm^–1^. This would result in theory in an ideal spectral resolution of 3.5 cm^–1^. Due to the spectrometers internal 1:1 imaging and the 300-µm-wide finite-sized pinhole, this value is reduced to approximately 40 cm^–1^.

The line array of the camera is working in full vertical binning mode, summing up the vertical pixels at 50 kHz horizontal pixel readout rate; and a pre-amplification gain of 1×. On average, these camera settings will result in 4.34 measured A/D-counts for every electron and therefore for every detected photon. Using the wavelength-dependent quantum efficiency *QE(λ)* of the camera the number of detected photons is calculated. The detected Raman scattering rate $R_{Raman}$ is used to quantify the signal strength:

(7) $$R_{Raman}=\;\frac{N_{e}}{QE\left(\lambda \right)\cdot t_{Int}}=\frac{C}{4.34\cdot QE\left(\lambda \right)\cdot t_{Int}}\;,$$

with *N_e_* being the number of excited electrons, $t_{Int}$ the integration time and *C* the number of A/D counts. The spatial mode inside the cavity is recorded using a camera (Spotlight Webcam Pro, Trust, Dordrecht, Netherlands). 

A Pound–Drever–Hall (PDH) lock is used to actively stabilize the cavity length to resonance of the ECDL’s wavelength and therefore to maximum internal intensity [75,76,77]. To this end, a modulation with a frequency $\nu_{mod}=80$ MHz is applied to the ECDL, adding two sidebands in the frequency spectrum. Mixing the back reflection from the incoupling mirror with the modulation frequency allows the PDH error signal to produce. The PDH electronics are custom built and are used to control the piezo in such a way that the cavity reflectivity is kept at its minimum, in turn leading to maximum intensity inside the cavity. 

The cavity is positioned in a gas-tight aluminum box with a volume of 10 L. Light is coupled into the box via a sapphire window of 8 mm in diameter placed in Brewster’s angle, to achieve high transmission with no intensity reflected. During gas measurements, a beam dump is placed on the backside of the cavity to reduce scattered photons entering the spectrometer. The gas composition inside the chamber is adjusted using the setup depicted in Figure 1. It is based on a setup to control the atmosphere in a test chamber [78]. A probe gas cylinder and a pure nitrogen gas cylinder with 200 bar internal pressure are regulated down with single stage pressure regulators to about 2 bar absolute output pressure. Then each is connected to a software controlled mass flow controller (FC 280 S, Tylan, San Diego, CA, USA) able to operate at gas flows of up to 2 L/min, hence able to adjust the concentrations of gases via the relative gas flows. The pressure inside the measurement chamber is 1 bar. During experiments a flow of 1.5 L/min has been employed. Both gas flows are mixed and put into the gas flow box via a M5 gas fitting (Quick Star straight, Festo, Esslingen, Germany). Four gases and their mixtures have been tested. The specific Raman shifts according to literature are stated in Table 1. All gases play an important role in food chain monitoring. All measurements are performed at an outlet pressure of approximately 1 bar at the exhaust and a temperature of 23 °C.

### 2.2. Simulation

The behavior of the system has been simulated and the models developed may be easily adopted to other cavity enhanced Raman setups. The simulation contains two parts: (1) Calculation of the spatial distribution of the pump light and (2) simulation of the spatial distribution of the Raman scattered light. 

**Figure 2:**
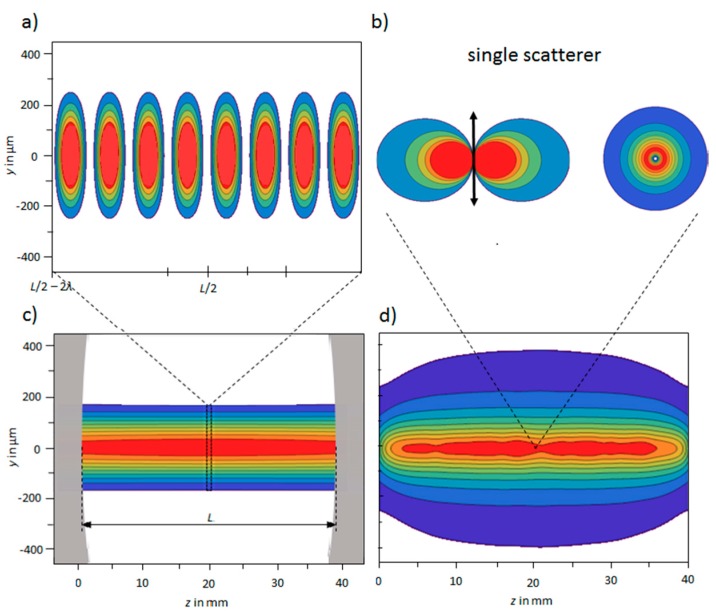
(**a**) Confined view of the laser intensity distribution inside the center of the cavity as a standing wave superimposed with a Gaussian beam and (**c**) full view of the intensity distribution over the cavities’ full length. The cavity mirrors are indicated in gray. (**b**) Emission characteristics of a single scatterer with respect to the dipole axis. (**Left**) is a sectional view through the scattering intensity with a plane spanned by the dipole axis, whereas (**right**) shows a full view from above the scatterer. (**d**) Simulated Raman scattering pattern of 20,000 particles inside the cavity at a distance of 10 mm in the *z-y* plane.

The intensity distribution is determined by the wavenumber *k*, the beam radius W(z), the beam waist $W_{0}$ and the the intensity $I_{0}$ in the focus of the beam forming a standing wave with a total of more than 10^5^ maxima for the currently employed cavity. The generated Raman intensity is proportional to the pump intensity distribution but the spatial distribution is overlapped by the directional characteristics of a Hertzian dipole for each individual scatterer. The induced dipole oscillates at the same frequency and in the same direction as the electric field of the excitation light [80]. The emitted intensity of such a spherical dipole scatterer reads [81]:

(8) $$I\left(r,\theta \right)\propto \frac{\sin{\left(\theta \right)}^{2}}{r^{2}}\;,$$

where the variable *r* defines the distance from the center of the scatterer and *θ* describes the angle to the dipole axis. In Figure 2 the characteristic of such a dipole as well as a cavity filled with scatterers is simulated. Therefore 20,000 molecules have been randomly placed inside the cavity and the resulting spatial distribution of Raman light has been visualized for a 40 × 10-mm²-sensor in a distance of 10 mm from the center. Based on this simulation the collection optics have been selected. 

## 3. Results and Discussions

The results are separated into the characterization of the Fabry–Pérot cavity and the gas sensitive characterization. Firstly, the resonator specific properties like resonator linewidth, free spectral range and Finesse are determined. Secondly, the gas measurements done with the Raman spectroscopy system for food chain gases are presented. 

### 3.1. Cavity Characterization

**Figure 3:**
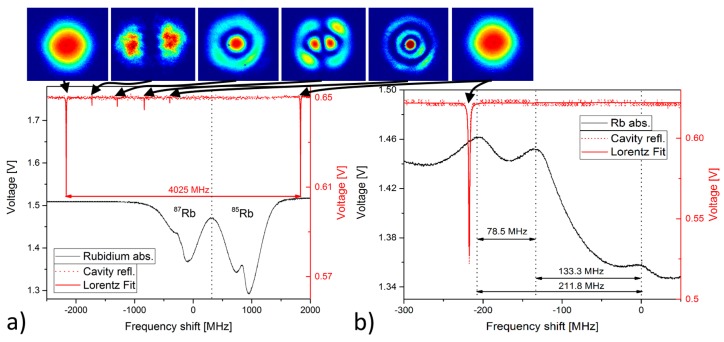
(**a**) Absorption spectrum of Rubidium (black), and simultaneously measured cavity reflection (red) as detected with photodiodes, while the frequency shift is achieved by tuning the wavelength of the ECDL at a scanning rate of 45 Hz. The intensity pattern above the graphs are recorded by a camera placed after the cavity and make the identification of the various excited TEM eigenmodes of the cavity possible. (**b**) Zoom into the R87b absorption band at another offset value of the cavity Piezo. A Lorentzian fit is applied to the cavity reflection to determine the linewidth of the cavity.

For calibration of the cavity its Piezo voltage is held constant and the wavelength of the ECDL is tuned to simultaneously record Doppler free Rb and cavity spectra near the D2 line of Rb [82,83] and both spectra are depicted in Figure 3. The ECDL exhibits a mode-hop free scanning range exceeding 4 GHz, which is sufficient for the current application. In turn the cavity Piezo allows for scanning >10 GHz. Using the well-known absolute frequencies of the Rb transitions and the involved cross-over transitions, the frequency axis is calibrated and the free spectral range is determined to be $\nu_{FSR}$ = (4.025 ± 0.003) GHz, recognizable by the separation between two ${\mathrm{TEM}}_{00}$-modes. Apart from the fundamental mode, several higher order Laguerre–Gaussian beams appear with resonance frequencies shifted by their respective Gouy phase shifts Δζ according to [73]:

(9) $$\nu_{q,l,m}=\;q\cdot \nu_{FSR}+\frac{\Delta \zeta }{\pi }\cdot \nu_{FSR}\left(l+m+1\right)$$

where (*l,m*) is the order of the Gaussian beam. 

A smaller scanning range at a second offset value of the cavity Piezo is shown in Figure 3b. The data is used to determine the cavity linewidth by applying a Lorentzian fit resulting in a full width at half maximum cavity linewidth of Δυ = (1.51 ± 0.02) MHz. This value is actually a convolution of the ECDL linewidth and the cavity linewidth and we neglect the ECDL influence on this result because its linewidth is in the kHz range [72]. The actual cavity hence exhibits a Finesse *F* = (2666 ± 47) at a slightly smaller than expected mirror distance of 37.24 mm and mirror reflectivity of (99.8825 ± 0.0020)% exceeding specifications. The corresponding quality factor exceeds Q=υ0Δυ≈2.5·108. The power coupled into the cavity *P_in_* is 9.3% of the total available power of 32 mW directly in front of the cavity resulting in 2.9 mW incoupled power, which has been determined using the transmitted power on resonance $P_{trans}=1/2\cdot P_{in}=1.45$ mW. Inside the resonator it is enhanced to the following internal power, where *R* is the mirrors reflectivity:

(9) Pges=∑n=1∞Pin·Rn=Pin(1−R)=(2.46±0.04) W ,

with a power enhancement factor A=1/(1−R)=(851±15).

### 3.2. Gas Measurements

**Figure 4:**
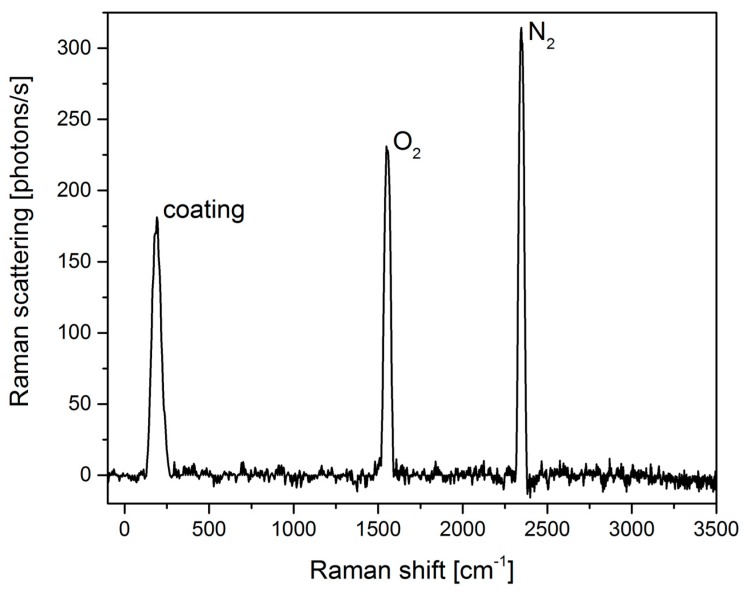
Raman spectrum for air with 10 s integration time with the measured Raman bands for oxygen, nitrogen and the coating material.

To determine the background signal of the cavity and check for possible malfunctions measurements using laboratory air are performed and the results are shown in Figure 4. Three Raman band positions can be identified with two of them related to oxygen at 1555 cm^−1^ and nitrogen at 2331 cm^−1^. The third peak at 214 cm^−1^ belongs to the high reflectivity dielectric coating’s crystalline surface [25] of the mirrors. Because the intracavity power vanishes quickly inside the mirrors a significant contribution to the signal is only expected from the first layer of the dielectric coating of the mirrors. 

**Figure 5:**
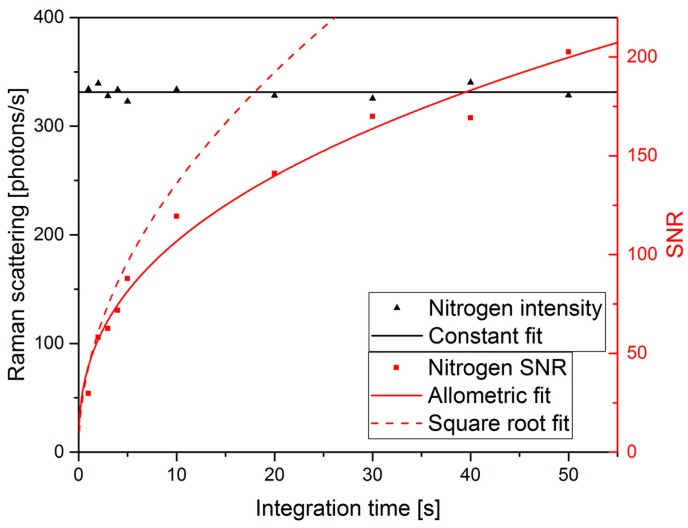
Raman scattering rate of ambient nitrogen, i.e., at a concentration of 79% at 1 bar, with a constant fit, next to the SNR of nitrogen at the varying integration times. The square root fit is showing the ideal progress of the SNR, if only limited by photonic noise. Whereas the allometric indicates the real progress.

Because we have opted for plotting the scattering rate, increases in the integration time results in a reduced background noise rather than higher peaks. Nonetheless, the signal-to-noise-ratio (SNR) improved and in Figure 5 ten values for the integration time are shown together with their corresponding nitrogen Raman scattering rate and the SNR at each position, ranging from 1 to 5 s in steps of 1 s and from 10 to 50 s in 10-s-steps. The signal S is defined as peak height and the noise is evaluated as the standard deviation σ of the background noise from 1000 cm^−1^ to 1200 cm^−1^ to determine the fluctuation of a single pixel. The SNR is calculated as SNR=Sσ. The nitrogen intensity of lab air corresponds to a mean 331.30 photons/s Raman scattering rate, with a standard deviation around the mean value of 5.43 photons/s or 1.64%. A constant fit shows that the Raman scattering rate is independent on the integration time, which highlights the long-term stability of the setup. The photonic noise follows a Poisson-like distribution, meaning that for N photons the noise is increasing with $\sqrt{N}$ [84], or in terms of increasing integration times with $\sqrt{t_{int}}$. Usually also signal intensity from other sources is detected, such as electronic noise, stray light or fluorescent light, and contributes to the noise ratio. In this experiment the CCD array is cooled to −70 °C, drastically reducing the electronic noise. Still stray light cannot be excluded from the background signal, which is why the SNR is fitted and evaluated with a fit of the form:

(10) $$y=a\cdot t_{int}^{b}\;,$$

delivering a=43.5±3.6 and b=0.390±0.025 as fit parameters visible in Figure 5 marked as allometric fit. In addition the ideal SNR development for the same a-value and b=0.5 is shown as square root fit in order to demonstrate that other noise sources must contribute to the measurement signal. Hence the improvement of the SNR with integration time is smaller than expected. Still it remains an easily implemented method to improve both SNR and the limit of detection (LOD). 

**Figure 6:**
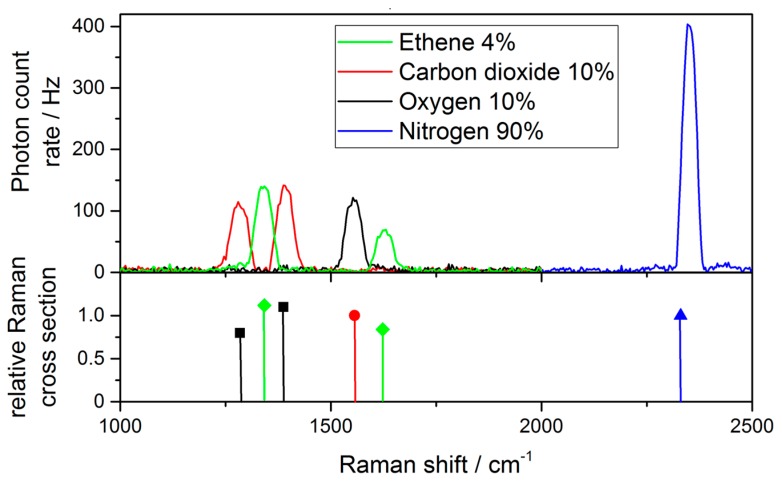
Relative Raman scattering cross sections of the climacteric gases as well as nitrogen at their respective Raman shifts, compared to their measured Raman scattering rates. Note that ethene is measured at 4% concentration and therefore the weighted scattering cross section for ethene is shown.

The gases tested in here can be distinguished by their specific Raman shift, which are listed in Table 1. To confirm the calibration of the spectrometer the theoretical Raman bands are compared with real measurements and the result is shown in Figure 6. The upper part of the Figure is showing measurements of the following gas concentrations: 4% ethene, 10% carbon dioxide, 10% oxygen and 90% nitrogen. The lower part displays the relative Raman scattering cross sections for ethene, carbon dioxide, oxygen and nitrogen at their corresponding wavelength shift. While the peaks at lower wavelength shifts are in good agreement with their theoretical values, this is not the case for the upper ethene peak (at 1623 cm^−1^) and for the nitrogen peak. A possible reason for the discrepancy is the chromatic dispersion of the detection lenses leading to a spatial shift of the focus of higher wavelengths, which then are partly blocked by the pinhole and not perfectly focused on the camera.

**Figure 7:**
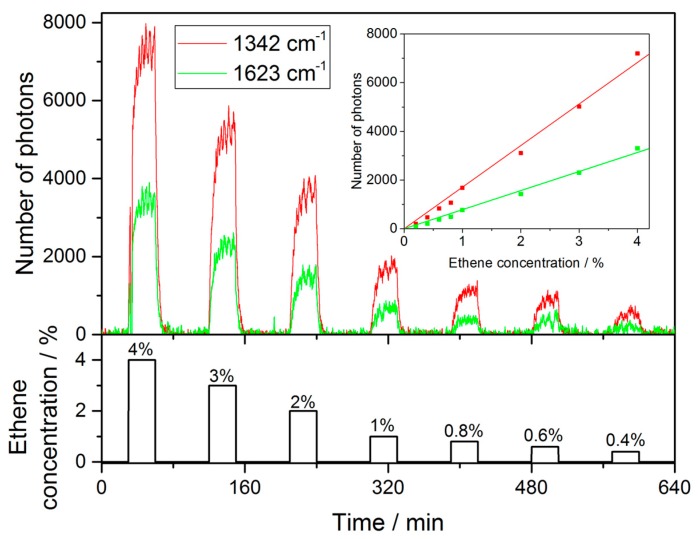
Signal of ethene, i.e., the peak height at 1342 cm^−^^1^ and 1623 cm^−^^1^, respectively, for different concentrations over time during a measurement. The time necessary to reach equilibrium inside the measurement chamber can be clearly observed. The number of scattered photons has been determined from an integration time of 30 s. Note that only the values of the respective camera pixels are plotted and not the background noise.

For a full understanding of the system it is also necessary to verify the proper behaviour to changes in concentrations. Therefore the system is exposed to several concentrations and a sample measurement using varying levels of ethene is depicted in Figure 7 highlighting the long-term stability of the cavity setup and its suitability for real-world deployments. In this instance the cavity has been working with a stable lock for more than 12 h and in the graph the pixel values corresponding to the Raman bands at 1342 cm^−^^1^ and 1623 cm^−^^1^ at an integration time of 30 s have been plotted. Due to the volume of the measurement chamber and the flow rate it takes about 20 min to reach a new steady state concentration upon a change of the gas composition. Hence the evaluation of the response towards a specific gas only takes into account readings after a new equilibrium has been established. 

**Figure 8:**
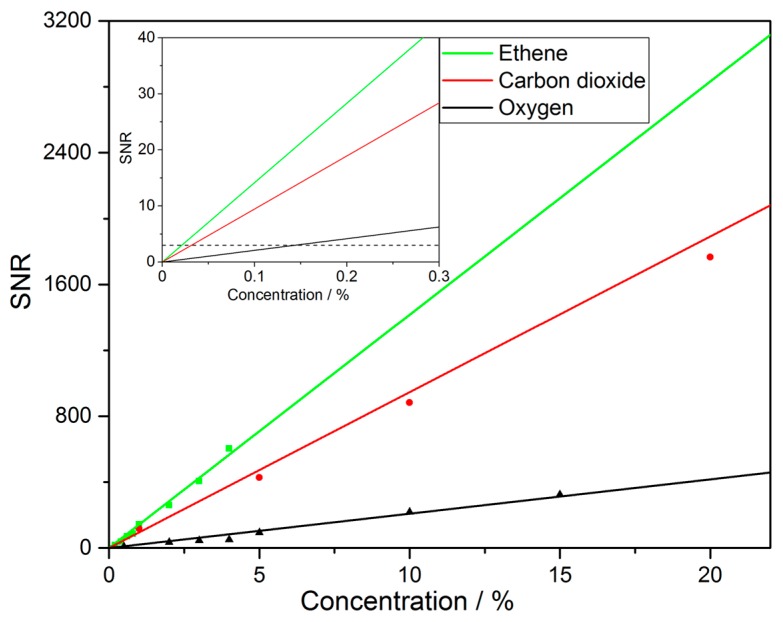
SNR of ethene, carbon dioxide and oxygen for different concentration steps at 30 s integration time. The limit of detection is defined as the concentration where SNR = 3 for each gas and the inset highlights this value as a dotted line.

Similar measurements have been performed for CO_2_ and O_2_. The analysis of the response is presented in Figure 8. The signal-to-noise ratio presented therein corresponds to an integration time of 30 s. Defining the limit of detection as SNR = 3 it may be derived from the analysis of this graph. Using a linear fit of the form $SNR\;=\;A\cdot c_{gas}$, where *A* is the sensitivity and $c_{gas}$ the concentration of the respective gas in ppm, the LOD has been determined and the results are summarized in Table 2. An improvement in LOD may be achieved via longer integration time scaling with $t_{int}^{0.39}$.

## 4. Conclusions

We have presented the design, characterization and operational capacity of a stable system to measure the gases relevant in food chain management. Using a Fabry–Pérot cavity, the power available to pump spontaneous Raman scattering is enhanced 851 fold. Using Doppler free Raman spectroscopy as frequency standard the characteristics of the enhancement cavity have been determined. The cavity linewidth is 1.51 MHz at free spectral range of 4.025 GHz resulting in an optical Finesse of 2666. With those details the mirror reflectivity is determined to be 99.8825% and the distance between the two mirrors is 37.24 mm. Coupling 9.6 mW into the cavity thus leads to an internal power of 8.17 W available for Raman scattering. Using this setup, it is possible to detect the most relevant gases in food chain management and in fact, the system does have a wide range of possible applications including biogas analysis, breath gas analysis or environmental monitoring. In particular, the detection capabilities are not limited to the four gases presented here but extend to all molecules that show rovibrational Raman spectra in the range up to 3500 cm^−1^, which includes water vapor, methane, hydrogen sulfide. Measurements show the influence of the integration time to reduce the influence of noise and the possibility to improve the limit of detection. The setup shows long term stability with a constant baseline over more than 12 h. The active stabilization delivers a constant power enhancement in the cavity and a thereby stable system to quantify the gases. While this contribution mostly devotes to the characterization of the system’s performance, it also has been able to demonstrate the potential of the cavity enhanced Raman spectroscopy approach. 

## Figures and Tables

**Table 1:** Raman shifts of nitrogen, oxygen, carbon dioxide and ethene gas. Listed with their relative scattering cross sections [28,79].

Gas	Raman Shift [cm^−1^]	Relative Cross Section
Oxygen	1555	1
Carbon dioxide	1285	0.8
	1388	1.1
Ethene	1342	2.8
	1623	2.1
Nitrogen	2331	1

**Table 2:** Linear fits for the determined SNR of oxygen, carbon dioxide and ethene, and the resulting LODs. Note that the LOD values stated here are determined for the spectrometer used in the current setup.

Gas	A [ppm^−1^]	LOD@*t_int_*= 30 s [ppm]
Oxygen	($21.24\pm 0.51)\times 10^{-4}$	1412 ± 28
Carbon dioxide	$\left(94.58\pm 2.47\right)\times 10^{-4}$	317 ± 8
Ethene	$\left(114.59\pm 4.16\right)\times 10^{-4}$	261 ± 9
Nitrogen	(8.46 ± 0.64)$\;\times 10^{-4}$	3540 ± 267
